# National monitoring and evaluation of eHealth: a scoping review

**DOI:** 10.1093/jamiaopen/ooz071

**Published:** 2020-03-20

**Authors:** Sidsel Villumsen, Julia Adler-Milstein, Christian Nøhr

**Affiliations:** 1 Center for Health Informatics and Technology, Maersk Mc-Kinney Moller Institute, University of Southern Denmark, Odense, Denmark; 2 Center for Clinical Informatics and Improvement Research, School of Medicine, University of California, San Francisco, California, USA

**Keywords:** medical informatics, program evaluation, review

## Abstract

**Objective:**

There has been substantial growth in eHealth over the past decade, driven by expectations of improved healthcare system performance. Despite substantial eHealth investment, little is known about the monitoring and evaluation strategies for gauging progress in eHealth availability and use. This scoping review aims to map the existing literature and depict the predominant approaches and methodological recommendations to national and regional monitoring and evaluation of eHealth availability and use, to advance national strategies for monitoring and evaluating eHealth.

**Methods:**

Peer-reviewed and grey literature on monitoring and evaluation of eHealth availability and use published between January 1, 2009, and March 11, 2019, were eligible for inclusion. A total of 2354 publications were identified and 36 publications were included after full-text review. Data on publication type (eg, empirical research), country, level (national or regional), publication year, method (eg, survey), and domain (eg, provider-centric electronic record) were charted.

**Results:**

The majority of publications monitored availability alone or applied a combination of availability and use measures. Surveys were the most common data collection method (used in 86% of the publications). Organization for Economic Co-operation and Development (OECD), European Commission, Canada Health Infoway, and World Health Organization (WHO) have developed comprehensive eHealth monitoring and evaluation methodology recommendations.

**Discussion:**

Establishing continuous national eHealth monitoring and evaluation, based on international approaches and recommendations, could improve the ability for cross-country benchmarking and learning. This scoping review provides an overview of the predominant approaches to and recommendations for national and regional monitoring and evaluation of eHealth. It thereby provides a starting point for developing national eHealth monitoring strategies.

## INTRODUCTION

eHealth adoption has been growing substantially with high expectations for resulting improvements in healthcare system performance.^1^ eHealth investment was motivated by the need to improve healthcare quality, clinical care processes, and patient safety. However, eHealth infrastructure has proven highly costly to procure and maintain.^2^^,^^3^

Given these large investments, there has been a demand for monitoring of resulting adoption, use, and impact.^3^^,^^4^ Monitoring enables an understanding of what works and what does not, thus guiding improvements in implementation and adoption.^3^ Longitudinal monitoring can provide valuable feedback for adjusting and improving implementation strategy and underlying policies but is often both costly, time-consuming, and highly complicated.^5^^,^^6^ The fact that it may take years for potential benefits and consequences to appear substantiates the complex nature of monitoring and evaluating eHealth.^6^^,^^7^ Although the importance of monitoring and evaluating is recognized and essential in formulating future eHealth policies, repeated monitoring of implementation progress of the policies are often scarce.^6^

In 2009, a comprehensive study was conducted by Empirica, on behalf of the European Commission, aiming “to collate and analyze existing eHealth monitoring and benchmarking sources in order to identify best practice in data gathering and to develop a framework for an EU-wide eHealth Benchmarking approach.”^8^ The report presents a comprehensive list of indicators and approaches. However, the eHealth landscape has progressed vastly in the past decade, thus calling for renewed methods for national monitoring.

We, therefore, created an overview of the current approaches and methodologies for national and regional monitoring and evaluating eHealth availability and use. With this scoping review, we aim to provide an overview of the current literature produced by researchers, organizations, or government bodies, and to assess the foci, methodology, and scope of monitoring and evaluating eHealth. The focus of this scoping study lies not in addressing the quality of the studies and obtaining ‘best evidence’,^9^ but in creating an overview of the monitoring and evaluation activities to advance national strategies for monitoring and evaluating eHealth.

## MATERIALS AND METHODS

This scoping review is based on the approach suggested by the Joanna Briggs Institute, adapted and developed from the five stages by Arksey and O’Malley^10^ and the enhancements proposed by Levac et al.^11^ The objective, inclusion criteria, and methods for the scoping review were presented in a protocol.^12^ The reporting of this scoping review follows the checklist and flow described in the PRISMA Extension for Scoping Reviews (PRISMA-ScR).^13^

### Identifying relevant studies

eHealth is “the application of information and communication technologies across the whole range of functions that affect the health sector and including products, systems, and services that go beyond simply Internet-based applications.”^14^ This scoping review was restricted to eHealth in primary and secondary care. The Joanna Briggs Institute methodology suggests that the scope of the review should balance feasibility and maintaining a broad and comprehensive approach.^15^ This led us to focus on the three most prominent domains within information and communication technology (ICT) in health: provider-centric electronic records, patient-centric electronic records, and health information exchange (HIE).^16^

The search strategy aimed to ensure the identification of both peer-reviewed publications providing quantitative and/or qualitative evidence on monitoring or evaluating eHealth at a national or regional level; and other publications, peer-reviewed or not, opinions or reports. The search targeted a number of potential sources including journal citation databases, bibliographic databases, and output from known centers of excellence and governments. The protocol for this scoping review contains further information on the preliminary search strategies.^12^ A search for published scoping reviews did not reveal any scoping reviews with similar aims (databases searched JBISRIR, PubMed, The Cochrane Library, CINAHL, SCOPUS, and Web of Science).

To identify original peer-reviewed publications, we used the databases PubMed, SCOPUS, and Web of Science. Further, a structured search for grey literature, such as national or organizational reports, was performed using the Canadian Agency for Drugs and Technologies in Health (CADTH) checklist for grey literature “Grey Matters”.^17^ Danish, Norwegian, and Swedish national bibliographic databases were searched to identify Scandinavian literature on the topic. In addition, an informal chain search was applied through reference lists of relevant publications. The structured search was divided into two sections:

- Monitoring or evaluating *availability* of eHealth
- Monitoring or evaluating *use* of eHealth

In the availability section, key search terms were *Monitoring and Evaluation*, *eHealth*, and *Availability*. Each key term had several synonymous sub-terms. When applicable, major terms were used (ie, MeSH terms). To ensure the detection of literature not yet indexed with major terms, free text search was used. We did not seek to identify the specific metrics used for monitoring (ie, indicators) as these metrics would be specific to the organizational setup of national health systems and context.

A full search strategy for a PubMed search can be found in Table 1. For further information on the search strategy, see Supplementary material.^18^ The structured literature search was performed last on March 11, 2019.

**Table 1.** Search strategy for PubMed

Monitoring and evaluation	eHealth	Availability	Combinations		
“Program Evaluation”[Mesh] “Benchmarking”[Mesh] “Process Assessment (Health Care)”[Mesh] “evaluation” “evaluating” “monitoring” “assessment” “benchmark” ”benchmarking”	“Medical Informatics Applications”[Mesh] “Medical Informatics”[Mesh] “Information Systems”[Mesh] “Medical Records Systems, Computerized”[Mesh] “Telemedicine”[Mesh] “Hospital Information Systems”[Mesh] “Health Information Management”[Mesh] “Telemedicine” “Electronic medical record” “Hospital information system” “Electronic patient record” “Health information management” “Medical informatics” “health information and communication technology”	“Healthcare Disparities”[Mesh] “Health Services Accessibility”[Mesh: NoExp] “Diffusion of Innovation”[Mesh: NoExp] “Accessibility” “Availability” “availabilities” “Disparity” “Disparities”	(((((((((“Health Information Management”[Mesh]) OR “Hospital Information Systems”[Mesh]) OR “Telemedicine”[Mesh]) OR “Medical Records Systems, Computerized”[Mesh]) OR “Information Systems”[Mesh]) OR “Medical Informatics”[Mesh]) OR “Medical Informatics Applications”[Mesh])) AND (((“Health Services Accessibility”[Mesh: NoExp]) OR “Diffusion of Innovation”[Mesh: NoExp]) OR “Healthcare Disparities”[Mesh])) AND (((“Benchmarking”[Mesh]) OR “Program Evaluation”[Mesh]) OR “Process Assessment (Health Care)”[Mesh])	((((((((((“benchmarking”) OR “benchmark”) OR “monitoring”) OR “assessment”) OR “evaluation”) OR “evaluating”)) OR (((“Benchmarking”[Mesh]) OR “Program Evaluation”[Mesh]) OR “Process Assessment (Health Care)”[Mesh]))) AND ((((((((“Telemedicine”) OR “electronic medical record”) OR “Hospital information system”) OR “medical informatics”) OR “Electronic patient record”) OR “Health information management”)) OR (((((((“Health Information Management”[Mesh]) OR “Hospital Information Systems”[Mesh]) OR “Telemedicine”[Mesh]) OR “Medical Records Systems, Computerized”[Mesh]) OR “Information Systems”[Mesh]) OR “Medical Informatics”[Mesh]) OR “Medical Informatics Applications”[Mesh]))) AND (((((((“Disparities”) OR “Disparity”) OR “Availability”) OR “availabilities”) OR “Accessibility”)) OR (((“Health Services Accessibility”[Mesh: NoExp]) OR “Diffusion of Innovation”[Mesh: NoExp]) OR “Healthcare Disparities”[Mesh]))	((((((((“Telemedicine”) OR “electronic medical record”) OR “Hospital information system”) OR “medical informatics”) OR “Electronic patient record”) OR “Health information management”)) AND ((((((“benchmarking”) OR “benchmark”) OR “monitoring”) OR “assessment”) OR “evaluation”) OR “evaluating”)) AND (((((“Disparities”) OR “Disparity”) OR “Availability”) OR “availabilities”) OR “Accessibility”)

### Study selection

An iterative approach to selecting literature was applied, entailing continuous assessment of eligibility criteria and the screening process. A literature directory was created in Mendeley (Mendeley, v.1.19.4, Mendeley Ltd)^19^ and publications selected for screening were imported to Covidence, a web-based program for assisting review studies.^20^ All publications were checked for duplicates in both Mendeley and Covidence and screened by title and abstract, applying eligibility criteria. Literature published or in press between January 1, 2009 and March 11, 2019 was eligible for inclusion. To enable a thorough understanding of the included publications, we included literature published in English or Scandinavian languages only. Literature was excluded if it (1) described only a single IT-system, (2) was from a developing country, (3) described eHealth applications in dentistry, education and training of healthcare personnel, tele-homecare, telemedicine, nursing homes, or long-term care facilities, (4) the full text was not available, (5) the publication was an undergraduate, MSc, or PhD dissertation, or (6) the publication was a book review. Following the screening on title and abstract, full-text review was performed to determine final inclusion in this scoping review. All screening was performed by dual-review and differences in the assessment were resolved through consensus.

All materials were categorized into one of three broad categories based on an approach described by Wong et al.^21^ modified to our context:

- *Category 1: Peer-reviewed study with empirical measures of availability/use.* Clear articulation of the methodological approach to monitoring or evaluating availability or use of eHealth at a national or regional level, covering design, data collection, analyses, and relevance.
- *Category 2: Non-* *peer-* *reviewed report with empirical measures of availability/use.* Reports by government or non-government, health associations, professional bodies, and centers of excellence. We included these because national monitoring data is intended for public/broad consumption and therefore often not submitted for peer-review.
- *Category 3: Methodology recommendations.* Material presenting comprehensive recommendations of methodology of national monitoring or evaluating availability and/or use of eHealth. Often non-peer-reviewed.

Based on the approach by Arksey and O’Malley^10^ and Meyer et al.,^8^ information on category (ie, category 1, 2, or 3), country source, level of scope (national or regional), publication year, methods for monitoring or evaluating (eg, survey), whether one-off or repeated data collection, primary purpose, and eHealth domain were entered in a data charting form, see Supplementary material.^18^

### Domains

A general issue when comparing systems and services across countries is the subtle differences in terminology and understanding of what constitutes, for example, an electronic health record. Collecting and comparing data on functionalities rather than systems is a method of overcoming these cultural differences.^22^ Having mapped the publications descriptively, a narrative analysis, anchored within an adaption of the “Categories of broadly defined ICT domains” developed by Organization for Economic Co-operation and Development (OECD),^16^ see Table 2, will elaborate on how the monitoring and evaluation activities relate to the ICT domains in the health sector.

**Table 2.** Categories of broadly defined ICT domains

Provider-centric electronic records	Patient-centric electronic records	Health information exchange
Entry of core patient data (eg, medication allergies, clinical problem list)	Viewing of clinical data (eg, test results)	Secure messaging between professionals
Decision support (eg, drug–drug alerts)	Supplementation of clinical data (eg, entering or modifying current medications)	Ordering and reporting of medications and lab tests with result receipt
Closed-loop medication administration	Appointment scheduling	Patient referrals
Clinical documentation	Medication renewal	

Based on OECD^16^		

Provider-centric electronic records cover the range of Electronic Medical Records (EMRs), Electronic Health Records (EHRs), and Electronic Patient Records (EPRs) and “include systems that are used by healthcare professionals to store and manage patient health information and data, and include functionalities that directly support the care delivery process.”^16^ The definition emphasizes that the users are healthcare professionals. From the patient perspective, the patient-centric electronic records cover systems and functionalities such as Personal Health Records (PHRs) and patient portals, providing access to health information and allowing patients and their informal carers to “manage their health information and organize their health care.”^16^ HIE is the necessary link between different systems and organizations. It is the “process of electronically transferring, or aggregating and enabling access to, patient health information and data across provider organizations.”^16^

## RESULTS

**Figure 1.**
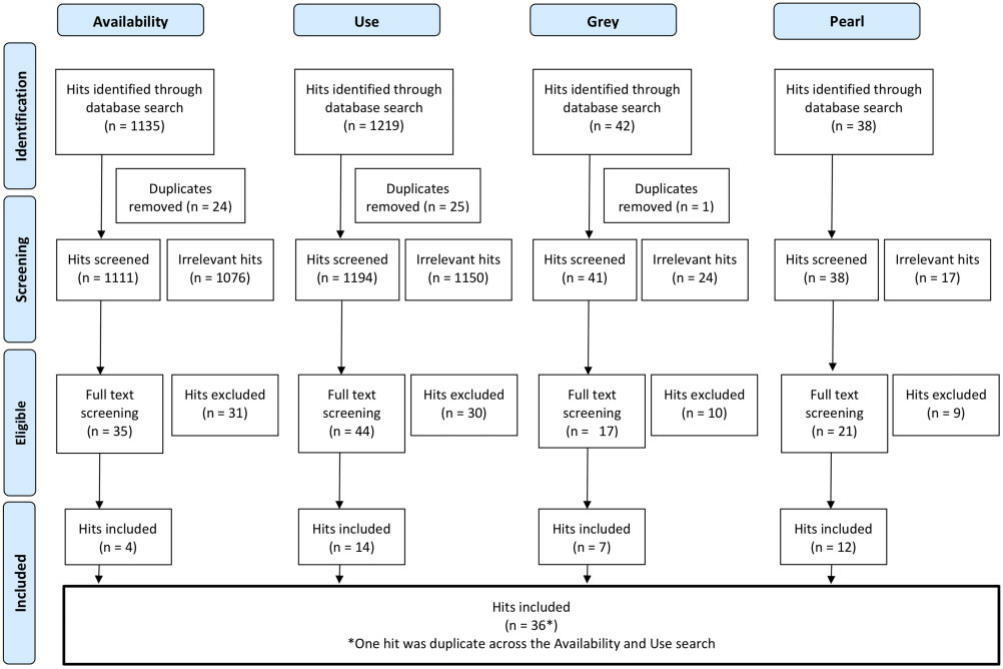
PRISMA flow chart.

The results of the search strategy provided a list of 1135 indexed publications for monitoring and evaluating eHealth availability and 1219 indexed publications for monitoring and evaluating eHealth use, see Figure 1. The grey literature search resulted in an additional 42 reports, and the informal search resulted in an additional 38 publications, including peer-reviewed original research, non-peer-reviewed papers, opinions, and reports.

A total of 117 full-text publications were reviewed with 80 excluded due to not fulfilling eligibility criteria after all (eg, single system or wrong setting) or not having a clear description of the methodology. After removing one additional duplicate, a total of 36 publications were included in this scoping review. Of the 36 publications, 64% were empirical research studies, 28% were reports by governments or organizations, and 8% were published recommendations of methodology. Table 3 provides an overview of the characteristics of the included publications. The full data charting form is available in the Supplementary material.^18^

**Table 3.** Characteristics of publications included in this scoping review

				*n*	%
Total number of publications identified				36	100%
Category	Category 1: Empirical research study			23	64%
	Category 2: Published reports			10	28%
	Category 3: Published recommendations of methodology			3	8%
Source	No country source (no data material)			3	8%
	Single country source (EU member states)			9	25%
	Single country source (non-EU member states)			13	36%
	Multinational sources			11	31%
	Of which	Covering 2–10 countries		6	55%
		Covering >10 countries		5	45%
Scope	National scope			30	83%
	Regional scope			4	11%
	Other			2	6%
Data collection methodology	Survey			31	86%
	Business process data			3	8%
	Other methods or no data gathering			6	17%
One-time or repeated	Continuous/repeated			15	42%
	Non-continuous/one-off activities			17	47%
	Other or no data gathering			4	11%
Primary purpose	Measuring eHealth/ICT availability and use			32	89%
	Of which focused on		Availability only	12	37.5%
			Use only	8	25%
			Availability and use	12	37.5%
	Evaluation			3	8%
	Other			1	3%
eHealth Domain^a^	Provider-centric electronic records			31	86%
	Patient-centric electronic records			16	44%
	Health information exchange			19	53%

^a^Each publication can cover more than one OECD domain. Published recommendations of methodology are noted as well.

**Figure 2.**
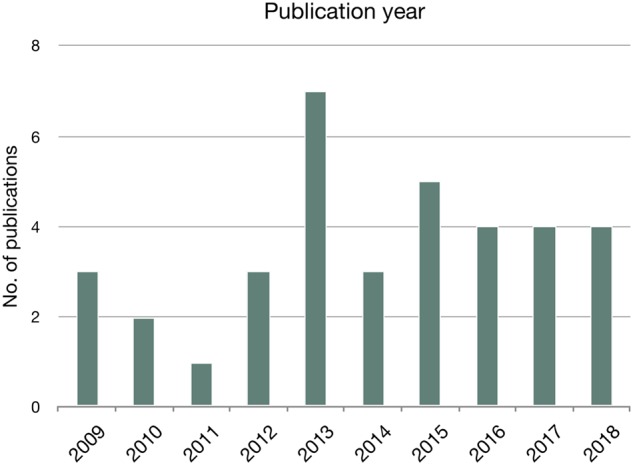
Distribution of publication year (*n* = 36).

Figure 2 shows the distribution of the publication year for the included publications. The distribution has been relatively evenly distributed throughout the period. The peak in 2013 reflects the Nordic eHealth Research Network reporting on the results of their mandate period^23^^,^^24^ as well as the OECD publishing their Guide to Measuring ICTs in the Health Sector.^16^

**Figure 3.**
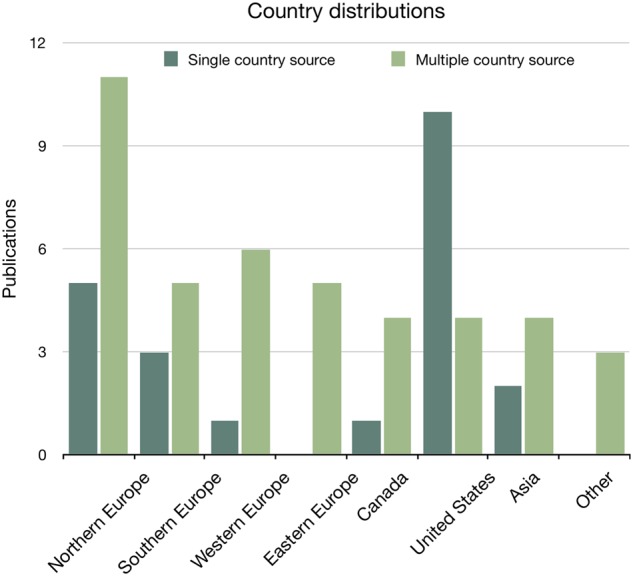
Distribution of publications on national monitoring and evaluating eHealth presented by single-country (*n* = 22) and multiple country sources (*n* = 11). Methodological recommendations are not included.

**Figure 4.**
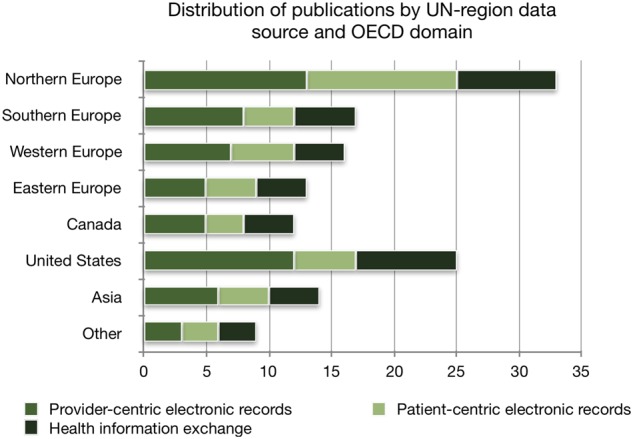
Distribution of publications by UN-region data source and OECD-domain (*n* = 33). Methodological recommendations are not included.

The geographical origin of monitoring and evaluating activities (Figure 3) is distributed across the United Nations’ definitions of regions (UN-region),^25^ with Northern European countries leading in multinational source publications and the United States leading on single-source publications. Figure 4 further shows the distribution of publications by UN-region data source and OECD-domain.

Mapping the publications showed that the majority of monitoring and evaluation activities were set in the Northern European countries and the United States. In 89% of the publications, the primary purpose was to monitor the availability and use of eHealth. Surveys were most commonly used (86% of the publications) and 42% of the publications referred to continuous or repeated data gathering activities.

### Provider-centric electronic records

*Publications: n = 28* ^23^,^24^,^26–51^

*Category:* 20 empirical studies^24^^,^^26–32^^,^^34–37^^,^^40^^,^^41^^,^^45–48^^,^^50^^,^^51^ and eight reports^23^^,^^33^^,^^38^^,^^39^^,^^42–44^^,^^49^

*Focus:* The main aims were on monitoring the availability and use of provider-centric electronic records or functionalities, for example, entering and viewing clinical data, medication list, picture archiving, and clinical decision support.^23^^,^^24^^,^^26–35^^,^^37–44^^,^^46–51^ Two focused mainly on evaluation, for example, the impact on the organization.^36^^,^^45^ Several publications explicitly aimed at presenting and testing a novel or modified methodology or approach to monitoring or evaluating.^23^^,^^24^^,^^28^^,^^31^^,^^40^^,^^45^^,^^47^

*Methods:* Most publications had a national scope (*n* = 24). Surveys were the data collection method most used to gauge the availability and use of provider-centric electronic records. Few publications also used business data (eg, log files) to measure availability and use of provider-centric electronic record functionality.^45^^,^^46^^,^^49^ Data collection was a mix of non-continuous (*n* = 18) and continuous or repeated activities (*n* = 10).

### Patient-centric electronic records

*Publications: n = 13* ^23^ ^,^ ^24^ ^,^ ^33^ ^,^ ^38–40^ ^,^ ^42^ ^,^ ^47^ ^,^ ^49^ ^,^ ^52–55^

*Category:* Five empirical studies^24^^,^^40^^,^^47^^,^^54^^,^^55^ and eight reports^24^^,^^33^^,^^38^^,^^39^^,^^42^^,^^49^^,^^52^^,^^53^

*Focus:* The main aims in all publications were on monitoring the availability and use of patient-centric electronic records or functionalities, for example, online appointment scheduling, medication renewal, viewing of clinical data, and electronic communication with General Practitioners. Four publications addressed the citizens’ perceptions of eHealth.^42^^,^^52^^,^^53^^,^^55^

*Methods:* All publications had a national scope and used surveys to gauge the availability and use of patient-centric electronic records. One publication also used business data (ie, log files) to assess the amount of use of patient-centric electronic record functionality.^49^ Data collection was a mix of non-continuous (*n* = 7) and continuous or repeated activities (*n* = 6). Only two publications surveyed patients directly^52^^,^^55^ and two publications used data already collected from citizen surveys performed in the Nordic Countries.^42^^,^^53^

### Health information exchange

*Publications: n = 16* ^23^ ^,^ ^24^ ^,^ ^32–34^ ^,^ ^37–42^ ^,^ ^45^ ^,^ ^46^ ^,^ ^48^ ^,^ ^49^ ^,^ ^56^

*Category:* 10 empirical studies^24^^,^^32^^,^^34^^,^^37^^,^^40^^,^^41^^,^^45^^,^^46^^,^^48^^,^^56^ and six reports^23^^,^^33^^,^^38^^,^^39^^,^^42^^,^^49^

*Focus:* The main aim was on monitoring the availability and use of HIE (*n* = 15), such as ePrecriptions, eReferrals, and exchange of clinical history, laboratory results, or radiology reports with external organizations. Only one publication regarded evaluating the systems’ effect on the organization.^45^

*Methods:* Most publications had a national scope (*n* = 13). Surveys were the data collection method most used to gauge the availability and use of HIE functionalities. A few publications also used business data (eg, log files).^45^^,^^46^^,^^49^ Data collection was a mix of non-continuous (*n* = 6) and continuous or repeated activities (*n* = 10).

### Methodological recommendations

*Publications: n = 3* ^8^ ^,^ ^16^ ^,^ ^57^

*Origin:* Canada Health Infoway,^57^ European Commission,^8^ OECD.^16^

*Focus:* The publications present thorough methodological recommendations and approaches to monitoring and evaluating eHealth. Methodological recommendations and a wide selection of indicators are provided within different domains and functionalities. All OECD domains presented in Table 2 are addressed. Canada Health Infoway focuses on benefits evaluation indicators,^57^ whereas the other publications aim at providing methodologies for cross-country benchmarking of eHealth availability and use.^8^^,^^16^

*Methods:* Data collection through survey methods is the main methodology described. Canada Health Infoway also emphasizes the use of business data (eg, log data and administrative data) and describes which indicators could be monitored by methods other than surveys.^57^ The methodology described in Canada Health Infoway focuses on national or regional evaluations,^57^ in contrast to the multinational scope of the European Commission and OECD.^8^^,^^16^

## DISCUSSION

This scoping review synthesizes the current literature on national approaches to monitoring and evaluation of availability and use of eHealth.

### Monitoring availability and use of eHealth

While availability and use are distinct concepts and of independent value to national measurement strategies, the literature reflects a lack of clear distinction between them. Many of the titles and abstracts of the publications indicate and state measurements of use (eg,^26^^,^^29^^,^^30^^,^^50^) but in fact, they monitor if services or functionalities are available to the users. Several publications report use as the *ability* to use a given functionality or system (eg,^26^^,^^29^^,^^30^^,^^50^) which is not the same as whether the functionality is actually being used and to which extent. It seems *adoption* as a term is often used when applying measures of availability of eHealth functionality as a proxy measure for actual use. This calls for a clearer distinction between monitoring the availability and the use of eHealth, as once saturation of availability is reached, use is the next step on the causal pathway to achieving impact.^46^^,^^58^ Hence, monitoring the actual use of a functionality, and whether it is used as intended, is a key element in evaluating the functionality and moving toward eHealth supporting clinical practice. Our study also reveals that only a few of the resources assessed national or regional eHealth impact as part of the monitoring strategy.^36^^,^^45^^,^^57^

### Some ICT domains better covered than others

The distribution of OECD domains covered in the included publications shows that provider-centric electronic records was by far the domain most often addressed (86% of all publications), whereas patient-centric electronic records were only addressed in 44% (Table 3). This could be ascribed to patient-centric electronic records being a relatively new point of focus,^52^ with no publications available before 2013. The focus on patient-centric electronic records varies among the regional distribution of the included publications. We found that the patient-centric domain is most frequently addressed in publications that include data from Northern European countries (Figure 4). This may be partly attributed to the Nordic countries’ focus on patient-oriented eHealth services.^53^ As eHealth evolves in complexity and coverage, focal points in monitoring and evaluating new functionalities and methods of doing so needs to be addressed. Thus, methodological approaches to monitoring and evaluating eHealth must be under continuous development.

### Methodological approaches and recommendations to monitoring and evaluating availability and use of eHealth

Surveys are by far the most common data gathering method for monitoring and evaluating national availability and use of eHealth (used in 86% of the publications). Surveys are cost-efficient and can be used to obtain information on phenomena that are not easily measured otherwise. However, surveys are prone to issues of low external validity and bias. Recall and social desirability biases are also common limitations of surveys.^59^ Using other sources of data that may be more objective, for example, log data, to monitor eHealth use, is a way to circumvent the drawbacks of surveys. Harvesting log data from central servers may be a reliable and valid approach.^5^ However, only three publications explicitly used such data,^45^^,^^46^^,^^49^ likely because needed centralized infrastructure does not exist and data on indicators of interest might not be logged in a manner that enables extraction. Furthermore, there is an issue of data ownership. Private vendors typically regard their data models as intellectual property and therefore do not want them to be made public, which may be needed to collect national-level data. A method of enhancing the possibilities of monitoring eHealth implementation through system-generated data is by defining indicators up-front and designing the data model of the systems in a way that allows for easy data extraction.^5^ Even so, there may be discrepancies between the clinical work routine and how it is captured by the system. Therefore, a prerequisite for analyzing and interpreting such data is knowledge of the context.

Our results also reveal the potential challenge of lack of repeated national monitoring and evaluation efforts. Repeated or continuous data collection is needed to measure secular progress or to evaluate the impact of policy changes (or other interventions). Ongoing measures of eHealth progress, therefore, supports evidence-based approaches to eHealth policy.^1^ We suspect that our finding that only 42% of the publications are part of or present data from continuous data gathering activities, such as annual or biannual surveys, reflects the time, resources, and complexity involved in large-scale data collection as well as changing national priorities. As previously described, building approaches to measurements relying on system-generated indicators could help increase the ability to pursue repeated measures.

Finally, our results reveal that, while there are national or international methodological approaches to eHealth monitoring, there are multiple approaches that are not harmonized.^22^ OECD, European Commission, and Canada Health Infoway have developed comprehensive approaches to eHealth monitoring and evaluation. The European Commission approach is only explicitly applied within the European Commission studies.^8^^,^^38^^,^^39^ Furthermore, WHO developed their own approach to international eHealth monitoring.^60^ The approach can be found applied in^39^ and,^38^ but since the report describing this was published in 2008,^60^ the report was not included in this scoping review. Finally, the OECD and Canadian methodological recommendations to monitoring and/or evaluating availability and use are more frequently applied. The Canadian approach, which focuses on benefits evaluation,^57^ and the OECD approach aiming at cross-country benchmarking,^16^ might be the most promising candidate methodologies for consistent national eHealth monitoring and evaluating.

### Limitations of this scoping review

The search strategy required iteration as the terminology within the research field of eHealth changed over the years, and it required adding new terms and definitions (eg, mHealth). In addition, many publications on eHealth monitoring and evaluation might only be disseminated through conferences or posters, which are not indexed in bibliographic databases in general. Thus, the choice of search terms and the focus on bibliographic databases may induce selection bias.

Most publications evaluated eHealth at the single institution or single-system level - and were therefore excluded. To capture a broader set of eHealth monitoring efforts, we included grey literature and it is possible that our results would be different if we had limited studies to the peer-reviewed literature. However, we do not feel that the peer-review process would fundamentally alter the content or methods of the monitoring that was the focus of our review.

## CONCLUSIONS

Monitoring eHealth adoption is essential for providing an evidence base on which to formulate future national eHealth policies and for evaluating the effectiveness of the efforts.^22^ Monitoring the adoption and impact of eHealth is key to learning from the past and current initiatives to provide evidence for decision-makers to base eHealth policy decisions upon.^1^ This scoping review provides an overview of the predominant approaches and methodological recommendations to national and regional monitoring and evaluation of eHealth. In order to establish an evidence base for eHealth policies, monitoring and evaluation should be continuous, allowing for trends and developments to unfold. Furthermore, applying a framework that allows for cross-country comparisons will broaden the evidence base of what works and what does not. The monitoring and evaluation activities should be transparent and published to facilitate benchmarking and learning. Implications for practice are to establish a governance structure around national eHealth monitoring, ensuring repeated and valid data on eHealth implementation progress.

## Author Contributors

The authors contributed to the manuscript as follows:

- Substantial contributions to the conception and design of the work (Villumsen and Nøhr); and the acquisition, analysis, or interpretation of data for the work (Villumsen, Adler-Milstein, and Nøhr).
- Drafting the work (Villumsen and Nøhr) and revising it critically for important intellectual content (Adler-Milstein).
- Final approval of the version to be published (all authors).
- Agreement to be accountable for all aspects of the work in ensuring that questions related to the accuracy or integrity of any part of the work are appropriately investigated and resolved (all authors).

## Supplementary Material

ooz071_Supplementary_Data

## References

[1] Hyppönen H, Ronchi E, Adler-Milstein J. Health care performance indicators for health information systems. Stud Health Technol Inform 2016; 222: 181–94. 27198102

[2] Cresswell KM, Sheikh A. Undertaking sociotechnical evaluations of health information technologies. Inform Prim Care 2014; 21: 78–83. 24841408 10.14236/jhi.v21i2.54

[3] Cusack CM, Byrne C, Hook JM, et al Health Information Technology Evaluation Toolkit: 2009 Update (Prepared for the AHRQ National Resource Center for Health Information Technology under Contract No. 290-04-0016). Rockville, MD: Agency for Healthcare Research and Quality; 2009.

[4] Flak LS, Solli-Saether H, Straub D. Towards a theoretical model for co-realization of IT value in government. *In: Proceedings of the 2015 48th Hawaii International Conference on System Sciences.* 2015; 2486–94; IEEE Computer Society.

[5] Villumsen S, Harðardóttir GA, Kangas M, et al Monitoring the amount of practical use of ehealth on national level by use of log data: lessons learned. Stud Health Technol Inform 2015; 218: 138–44. 26262541

[6] Cresswell KM, Bates DW, Sheikh A. Ten key considerations for the successful implementation and adoption of large-scale health information technology. J Am Med Informatics Assoc 2013; 20 (e1): E9–13. 10.1136/amiajnl-2013-001684 PMC3715363 23599226

[7] Ward J, Daniel E. Benefits Management: How to Increase the Business Value of Your IT Projects. Chichester, West Sussex: Wiley; 2012.

[8] Meyer I, Hüsing T, Didero M, et al eHealth Benchmarking (Phase II)—Final report. Bonn; 2009 https://joinup.ec.europa.eu/sites/default/files/files_epractice/sites/eHealth Benchmarking (Phase II)-Final Report.pdf (Accessed January 5, 2017).

[9] Slavin RE. Best evidence synthesis: an intelligent alternative to meta-analysis. J Clin Epidemiol 1995; 48 (1): 9–18. 7853053 10.1016/0895-4356(94)00097-a

[10] Arksey H, O'Malley L. Scoping studies: towards a methodological framework. Int J Soc Res Methodol 2005; 8 (1): 19–32.

[11] Levac D, Colquhoun H, O’Brien KK. Scoping studies: advancing the methodology. Implement Sci 2010; 5: 69. 20854677 10.1186/1748-5908-5-69 PMC2954944

[12] Villumsen S, Nøhr C. National monitoring and evaluation of health IT: protocol for a scoping review. Stud Health Technol Inform 2017; 234: 352–7. 28186067

[13] Tricco AC, Lillie E, Zarin W, et al PRISMA extension for scoping reviews (PRISMA-ScR): checklist and explanation. Ann Intern Med 2018; 169 (7): 467. 30178033 10.7326/M18-0850

[14] Codagnone C, Lupiañez-Villanueva F. A Composite Index for the Benchmarking of eHealth Deployment in European Acute Hospitals Distilling Reality in Manageable Form for Evidence Based Policy. Luxenbourg: European Commission—Joint Research Centre—Institute for Prospective Technological Studies; 2011.

[15] The Joanna Briggs Institute. Joanna Briggs Institute Reviewers’ Manual: 2015 edition/Supplement. 2015 www.joannabriggs.org (Accessed February 28, 2019).

[16] OECD Directorate for Employment Labour and Social affairs, OECD Directorate for Science Technology and Industry. Draft OECD guide to measuring ICRs in the health sector. 2013 https://www.oecd.org/health/health-systems/Draft-oecd-guide-to-measuring-icts-in-the-health-sector.pdf.

[17] Canadian Agency for Drugs and Technologies in Health. Grey Matters: a practical tool for searching health-related grey literature. Grey Matters a Pract. tool Search. Heal. grey Lit. 2015 https://www.cadth.ca/resources/finding-evidence/grey-matters (Accessed January 6, 2017).

[18] Villumsen S, Adler-Milstein J, Nøhr C. Data from: National monitoring and evaluation of eHealth: a scoping review. *Dryad Digital Repository* 2020; 10.5061/dryad.mk16b7r PMC7309231 32607495

[19] Mendeley Ldt. Mendeley. 2016 https://www.mendeley.com.

[20] Covidence. Covidence. 2016 https://www.covidence.org/.

[21] Wong MC, Yee KC, Turner P. Clinical Handover Literature Review. eHealth Services Research Group, University of Tasmania, Australia. 2008.

[22] Adler-Milstein J, Ronchi E, Cohen GR, et al Benchmarking health IT among OECD countries: better data for better policy. J Am Med Inform Assoc 2014; 21 (1): 111–6. 23721983 10.1136/amiajnl-2013-001710 PMC3912720

[23] Hyppönen H, Faxvaag A, Gilstad H, et al Nordic eHealth Indicators. TemaNord *2013:522*.

[24] Hyppönen H, Faxvaag A, Gilstad H, et al Nordic eHealth indicators: organisation of research, first results and plan for the future. Stud Health Technol Inform 2013; 192: 273–7. 23920559

[25] United Nations—Department of Economic and Social Affairs. Definitions of regions. 2011 https://esa.un.org/unmigration/Definition of regions.html. Accessed May 21, 2019.

[26] Kim YG, Jung K, Park YT, et al Rate of electronic health record adoption in South Korea: a nation-wide survey. Int J Med Inform 2017; 101: 100–7. 28347440 10.1016/j.ijmedinf.2017.02.009

[27] Kushniruk A, Kaipio J, Nieminen M, et al Comparing approaches to measuring the adoption and usability of electronic health records: lessons learned from Canada, Denmark and Finland. Stud Health Technol Inform 2013; 192: 367–71. 23920578

[28] Liebe JD, Hübner U. Developing and trialling an independent, scalable and repeatable it-benchmarking procedure for healthcare organisations. Methods Inf Med 2013; 52: 360–9. 23877646 10.3414/ME12-02-0016

[29] Marca G, Pérez AJ, Blanco-García MG, et al The use of electronic health records in Spanish hospitals. Heal Inf Manag J 2014; 43: 37–44. 10.1177/183335831404300305 27009795

[30] Nakamura MM, Harper MB, Jha AK. Change in adoption of electronic health records by US children’s hospitals. Pediatrics 2013; 131 (5): e1563–75. 23589808 10.1542/peds.2012-2904

[31] Palacio C, Harrison JP, Garets D. Benchmarking electronic medical records initiatives in the US: a conceptual model. J Med Syst 2010; 34 (3): 273–9. 20503611 10.1007/s10916-008-9238-5

[32] Park Y-T, Han D. Current Status of Electronic Medical Record Systems in Hospitals and Clinics in Korea. Healthc Inform Res 2017; 23 (3): 189–98. 28875054 10.4258/hir.2017.23.3.189 PMC5572523

[33] PwC. European Hospital Survey—Benchmarking Deployment of eHealth Services (2012-2013). Luxembourg: Publications Office of the European Union; 2014.

[34] Soderberg K, Laventure M. Minnesota clinics’ adoption, use and exchange of electronic health information. Minn Med 2013; 96 (9): 45–8. 24494362

[35] Viitanen J, Hyppönen H, Laaveri T, et al National questionnaire study on clinical ICT systems proofs: physicians suffer from poor usability. Int J Med Inform 2011; 80 (10): 708–25. 21784701 10.1016/j.ijmedinf.2011.06.010

[36] Buccoliero L, Calciolari S, Marsilio M, et al Picture, archiving and communication system in the Italian NHS: a primer on diffusion and evaluation analysis. J Digit Imaging 2009; 22 (1): 34–47. 18293039 10.1007/s10278-007-9101-0 PMC3043674

[37] Villalba-Mora E, Casas I, Lupianez-Villanueva F, et al Adoption of health information technologies by physicians for clinical practice: the Andalusian case. Int J Med Inform 2015; 84 (7): 477–85. 25823578 10.1016/j.ijmedinf.2015.03.002

[38] WHO Global Observatory for eHealth. Atlas of eHealth country profiles 2015: The use of eHealth in support of universal health coverage. Based on the findings of the 2015 global survey on eHealth. Geneva; 2016.

[39] WHO Global Observatory for eHealth. Global diffusion of eHealth: making universal health coverage achievable. Report of the third global survey on eHealth Global Observatory for eHealth. Geneva; 2016.

[40] Zelmer J, Ronchi E, Hyppönen H, et al International health IT benchmarking: learning from cross-country comparisons. J Am Med Inform Assoc 2017; 24 (2): 371–9. 27554825 10.1093/jamia/ocw111 PMC7651944

[41] Singh R, Lichter MI, Danzo A, et al The adoption and use of health information technology in rural areas: results of a national survey. J Rural Heal 2012; 28 (1): 16–27. 10.1111/j.1748-0361.2011.00370.x 22236311

[42] Hyppönen H, HäMäLäInen P, Reponen J. E-health and e-welfare of Finland. Tampere; 2015.

[43] Tornbjerg K, Nøhr C. Undersøgelse af klinisk anvendelse af sundheds-it-systemer 2014. Danish Centre for Health Informatics, Aalborg; 2014.

[44] HIMMS Analytical. Annual European eHealth Survey 2018. 2018 www.himss.eu/analytics (Accessed March 8, 2019).

[45] Colicchio TK, Del Fiol G, Scammon DL, et al Comprehensive methodology to monitor longitudinal change patterns during EHR implementations: a case study at a large health care delivery network. J Biomed Inform 2018; 83: 40–53. 29857137 10.1016/j.jbi.2018.05.018

[46] Gheorghiu B, Hagens S. Measuring interoperable EHR adoption and maturity: a Canadian example. BMC Med Inform Decis Mak 2016; 16 (8):doi:10.1186/s12911-016-0247-x. 10.1186/s12911-016-0247-x PMC4727402 26810606

[47] Haux R, Ammenwerth E, Koch S, et al A brief survey on six basic and reduced eHealth indicators in seven countries in 2017. Appl Clin Inform 2018; 9 (3): 704–13. 10.1055/s-0038-1669458 PMC6125136 30184560

[48] Hogan SO, Kissam SM. Measuring meaningful use. Health Aff 2010; 29 (4): 601–6. 10.1377/hlthaff.2009.1023 20368588

[49] Hyppönen H, Kangas M, Reponen J, et al. Nordic eHealth benchmarking—status 2014. TemaNord 2015:539.

[50] Jha AK, DesRoches CM, Campbell EG, et al Use of Electronic Health Records in U.S. Hospitals. N Engl J Med 2009; 360 (16): 1628–38. 19321858 10.1056/NEJMsa0900592

[51] Jaana M, Ward MM, Bahensky JA. EMRs and clinical IS implementation in hospitals: a statewide survey. J Rural Heal 2012; 28 (1): 34–43. 10.1111/j.1748-0361.2011.00386.x 22236313

[52] Petersen LS, Bertelsen P, Tornberg K. Undersøgelse af borgernes perspektiv på sundheds-it i 2015 - en udforskning af danskernes kendskab, holdninger, anvendelse og forhold til it til gavn for eget helbred. Danish Centre for Health Informatics. Aalborg Universitet. Technical Report No. 16–3.

[53] Hyppönen H, Kangas M, Reponen J, et al. Nordic eHealth Benchmarking—from piloting towards established practice.TemaNord 2017:528.

[54] Greenberg AJ, Haney D, Blake KD, et al Differences in access to and use of electronic personal health information between rural and urban residents in the United States. J Rural Heal 2018; 34: s30–8. 10.1111/jrh.12228 PMC5505819 28075508

[55] Bertelsen P, Stub Petersen L. Danish citizens and general practitioners’ use of ICT for their mutual communication. Stud Health Technol Inform 2015; 216: 376–9. 26262075

[56] Adler-Milstein J, Lin SC, Jha AK. The number of health information exchange efforts is declining, leaving the viability of broad clinical data exchange uncertain. Health Aff (Millwood) 2016; 35 (7): 1278–85. 27385245 10.1377/hlthaff.2015.1439

[57] Canada Health Infoway. Benefits Evaluation Indicators—Technical Report. Canada: Canada Health Infoway; 2012 https://www.infoway-inforoute.ca/index.php/programs-services/benefits-evaluation.

[58] Villumsen S, Faxvaag A, Nøhr C. Development and progression in Danish eHealth policies: towards evidence-based policy making. Stud Health Technol Inform 2019; 264: 1075–9. 31438090 10.3233/SHTI190390

[59] Althubaiti A. Information bias in health research: Definition, pitfalls, and adjustment methods. J Multidiscip Healthc 2016; 9: 211–7. 27217764 10.2147/JMDH.S104807 PMC4862344

[60] Scott RE, Saeed A. *Global eHealth—Measuring Outcomes: Why, What, and How A Report Commissioned by the World Health Organization’s Global Observatory for eHealth* Making the eHealth Connection website. Bellagio, Italy; 2008.

