# Supplementary material for: National monitoring and evaluation of eHealth: a scoping review
Source: JAMIA Open. 2020 Mar 20;3(1):132–40. doi: 10.1093/jamiaopen/ooz071 (PMC7309231; doi:10.1093/jamiaopen/ooz071)
Supplement: ooz071_Supplementary_Data [file ooz071_supplementary_data.zip › ooz071-Suppl_Data/Online supplementary Literature search strategy.pdf]

Search strategy

Database	Search	Search string	Filters and constraints
PubMed	Availability	(((((("Hospital Information Systems"[Mesh]) OR "Health Information Management"[Mesh])) OR ((((("Hospital information system"[Text Word]) OR "Health information management"[Text Word]) OR "medical informatics"[Text Word]) OR "Health informatics"[Text Word]) OR "Health information technology"[Text Word]))) AND (((((((("benchmarking"[Text Word]) OR "benchmark"[Text Word]) OR "assessment"[Text Word]) OR "monitoring"[Text Word]) OR "evaluating"[Text Word]) OR "evaluation"[Text Word])) OR (("Evaluation Studies"[Publication Type]) OR "Benchmarking"[Mesh]))) AND (((("Diffusion"[Text Word]) OR "Availability"[Text Word])) OR "Diffusion of Innovation"[Mesh:noexp])	
	Use	Search ((((((("use"[Text Word]) OR "adoption"[Text Word]) OR "Adoption of health it"[Text Word]))) AND (((((((("benchmarking"[Text Word]) OR "benchmark"[Text Word]) OR "assessment"[Text Word]) OR "monitoring"[Text Word]) OR "evaluating"[Text Word]) OR "evaluation"[Text Word])) OR (("Evaluation Studies"[Publication Type]) OR "Benchmarking"[Mesh]))) AND (((("Hospital Information Systems"[Mesh]) OR "Health Information Management"[Mesh])) OR ((((("Hospital information system"[Text Word]) OR "Health information management"[Text Word]) OR "medical informatics"[Text Word]) OR "Health informatics"[Text Word]) OR "Health information technology"[Text Word])))	

Database	Search	Search string	Filters and constraints
Scopus	Availability	((((TITLE-ABS-KEY (*"evaluation"*)) OR (TITLE-ABS-KEY (*"evaluating"*)) OR (TITLE-ABS-KEY (*"monitoring"*)) OR (TITLE-ABS-KEY (*"assessment"*)) OR (TITLE-ABS-KEY (*"benchmark"*)) OR (TITLE-ABS-KEY (*"benchmarking"*)))) AND (((((TITLE-ABS-KEY (*"Hospital information system"*)) OR (TITLE-ABS-KEY (*"Health information management"*)) OR (TITLE-ABS-KEY (*"Medical informatics"*)))) OR (((TITLE-ABS-KEY (*"Health informatics"*)) OR (TITLE-ABS-KEY (*"health information and communication technology"*)) OR (TITLE-ABS-KEY (*"Health information technology"*)))))) AND (((TITLE-ABS-KEY (*"Diffusion"*)) OR (TITLE-ABS-KEY (*"Availability"*)))))	
	Use	((((((TITLE-ABS-KEY (*"evaluation"*)) OR (TITLE-ABS-KEY (*"evaluating"*)) OR (TITLE-ABS-KEY (*"monitoring"*)) OR (TITLE-ABS-KEY (*"assessment"*)) OR (TITLE-ABS-KEY (*"benchmark"*)) OR (TITLE-ABS-KEY (*"benchmarking"*)))) AND (((((TITLE-ABS-KEY (*"Hospital information system"*)) OR (TITLE-ABS-KEY (*"Health information management"*)) OR (TITLE-ABS-KEY (*"Medical informatics"*)))) OR (((TITLE-ABS-KEY (*"Health informatics"*)) OR (TITLE-ABS-KEY (*"health information and communication technology"*)) OR (TITLE-ABS-KEY (*"Health information technology"*)))))) AND (((TITLE-ABS-KEY (*"adoption"*)) OR (TITLE-ABS-KEY (*"use"*)) OR (TITLE-ABS-KEY (*"adoption of health it"*)))))) AND (((TITLE-ABS-KEY (*"national"*)) OR (TITLE-ABS-KEY (*"regional"*))))	

Database	Search	Search string	Filters and constraints
Web Of Science	Availability	((“evaluation” OR "evaluating" OR "monitoring" OR ”assessment” OR "benchmark" OR ”benchmarking”) AND ("Hospital information system" OR "Health information management" OR "Medical informatics" OR ”Health informatics” OR "health information and communication technology" OR "Health information technology") AND (”Diffusion” OR "Availability"))	Time span: 2009-2019
	Use	(((“evaluation” OR "evaluating" OR "monitoring" OR ”assessment” OR "benchmark" OR ”benchmarking”) AND ("Hospital information system" OR "Health information management" OR "Medical informatics" OR ”Health informatics” OR "health information and communication technology" OR "Health information technology") AND ((("Adoption of health it") OR "use") OR "adoption") AND (”national” OR ”regional”)))	Time span: 2009-2019
Bibliotek.dk	Availability	(((tilgængelig*)) or ((udbredelse*))) and (((esundhed*)) or ((”sundheds-IT”)) or ((”sundheds and IT”)) or ((sundhedsinformati*)) or ((informationsteknologi*))) and (((benchmark*)) or ((sammenlign*)) or ((evaluering*)) or ((monitorer*)))<br><br>(((benchmark*)) or ((sammenlign*)) or ((evaluering*)) or ((monitorer*))) and ((((informationsteknologi*)) and ((sundhed*))) or ((esundhed*)) or ((”sundheds-IT”)) or ((”sundheds and IT”)) or ((sundhedsinformati*))) and (((tilgængelig*)) or ((udbredelse*)))	
	Use	((((informationsteknologi*)) and ((sundhed*))) or ((esundhed*)) or ((”sundheds-IT”)) or ((”sundheds and IT”)) or ((sundhedsinformati*))) and (((benchmark*)) or ((sammenlign*)) or ((evaluering*)) or ((monitorer*))) and (((anvendelse*)) or ((brug*)))<br><br>(((anvendelse*)) or ((brug*))) and (((esundhed*)) or ((”sundheds-IT”)) or ((”sundheds and IT”)) or ((sundhedsinformati*)) or ((informationsteknologi*))) and (((benchmark*)) or ((sammenlign*)) or ((evaluering*)) or ((monitorer*)))	

Database	Search	Search string	Filters and constraints
Bibsys.no	Availability	(helse AND (informasjonsteknologi* OR "informasjons- og kommunikasjonsteknologi*")) OR ”e-helse” AND tilgjengelig* OR formidling* OR spre	
	Use	(benchmark* OR sammenlign* OR evaluering* OR overvåking*) AND ((helse AND (informasjonsteknologi* OR "informasjons- og kommunikasjonsteknologi*")) OR ”e-helse”) AND (bruk*)	
Libris (kb.se/libris)	Availability	benchmark* OR övervakning* OR utvärdering* OR jämförelse* OR bedömning* AND ehälsa OR ((digital* OR informationsteknologi*) AND hälsa*)	
	Use	ehälsa OR ((digital* OR informationsteknologi*) AND hälsa*) AND utnyttja* OR använding*	
**Date of literature search update: March 11th, 2019**			
