# Supplementary material for: National monitoring and evaluation of eHealth: a scoping review
Source: JAMIA Open. 2020 Mar 20;3(1):132–40. doi: 10.1093/jamiaopen/ooz071 (PMC7309231; doi:10.1093/jamiaopen/ooz071)
Supplement: ooz071_Supplementary_Data [file ooz071_supplementary_data.zip › ooz071-Suppl_Data/Online supplementary Data chart.pdf]

Data chart

First Author	Publication year	Citation	Category	Country Source	Countries providing data	Region setting (UN)	Scope (National or Regional)	Data Source	Data Gathering	Main Purpose	ICT-Domain
Adler-Milstein	2016	Adler-Milstein J, Lin SC, Jha AK. The Number Of Health Information Exchange Efforts Is Declining, Leaving The Viability Of Broad Clinical Data Exchange Uncertain. *Health Aff (Millwood)* 2016;**35**:1278–85. doi:10.1377/hlthaff.2015.1439	1	Single country souce (non-EU)	USA	USA	National	Surveys	Continouing data gathering activities	Measuring eHealth/ict availability or use	HIE
Bertelsen	2015	Bertelsen P, Stub Petersen L. Danish Citizens and General Practitioners' Use of ICT for their Mutual Communication. *MEDINFO 2015 eHealth-enabled Heal* 2015;**216**:376–9. doi:10.3233/978-1-61499-564-7-376	1	Single country source (EU)	Danmark	Northern Europe	National	Surveys	Continouing data gathering activities	Measuring eHealth/ict availability or use	Patient
Buccoliero	2009	Buccoliero L, Calciolari S, Marsilio M, *et al.* Picture, archiving and communication system in the Italian NHS: a primer on diffusion and evaluation analysis. *J Digit Imaging* 2009;**22**:34–47. doi:10.1007/s10278-007-9101-0	1	Single country source (EU)	Italy	Southern Europe	National	Surveys	Non-continuous/ one off activity	Evaluation of eHealth applications/ services	Provider
Canada Health Infoway	2012	Canada Health Infoway. Benefits Evaluation Indicators - Technical Report. Canada: : Canada Health Infoway 2012. https://www.infoway-inforoute.ca/index.php/programs-services/benefits-evaluation	3	No data	No data	No data	No data	No data	No data	Evaluation of eHealth applications/ services	Provider, Patient, HIE
Colicchio	2018	Colicchio TK, Del Fiol G, Scammon DL, *et al.* Comprehensive methodology to monitor longitudinal change patterns during EHR implementations: a case study at a large health care delivery network. *J Biomed Inform* 2018;**83**:40–53. doi:10.1016/j.jbi.2018.05.018	1	Single country souce (non-EU)	USA	USA	Regional	Bussiness process data	Non-continuous/ one off activity	Evaluation of eHealth applications/ services	Provider, HIE
Gheorghiu	2016	Gheorghiu B, Hagens S. Measuring interoperable EHR adoption and maturity: A Canadian example. *BMC Med Inform Decis Mak* 2016;**16**. doi:10.1186/s12911-016-0247-x	1	Single country souce (non-EU)	Canada	Canada	National	Other	Continouing data gathering activities	Measuring eHealth/ict availability or use	Provider, HIE
Greenberg	2018	Greenberg AJ, Haney D, Blake KD, *et al.* Differences in Access to and Use of Electronic Personal Health Information Between Rural and Urban Residents in the United States. *J Rural Heal* 2018;**34**:s30–8. doi:10.1111/jrh.12228	1	Single country souce (non-EU)	USA	USA	National	Surveys	Continouing data gathering activities	Measuring eHealth/ict availability or use	Patient
Haux	2018	Haux R, Ammenwerth E, Koch S, *et al.* A Brief Survey on Six Basic and Reduced eHealth Indicators in Seven Countries in 2017. *Appl Clin Inform* Published Online First: 2018. doi:10.1055/s-0038-1669458	1	Multinational sources	Austria, Finland, Germany, Hong Kong, South Korea, Sweden, USA	Northern Europe, Western Europe, USA, Asia	National	Surveys	Non-continuous/ one off activity	Measuring eHealth/ict availability or use	Provider, Patient
HIMMS	2018	HIMMS Analytical. Annual European eHealth Survey 2018. 2018. www.himss.eu/analytics (accessed 8 Mar 2019).	2	Multinational sources	Denmark, Finland, Iceland, Norway, Sweden<br>"Others" include: Albania, Bulgaria, Croatia, Cyprus, Czech Rep., Estonia, Greece, Iceland, Lithuania, Malta, Poland, Russia, Slovakia, Slovenia, Ukraine, Turkey.<br>Germany Spain<br>Netherlands<br>Switzerland<br>Italy<br>Sweden Austria<br>Ireland<br>Belgium<br>France<br>Portugal<br>United Kingdom	Northern Europe, Western Europe, Eastern Europe, Southern Europe,	National	Survey	Continouing data gathering activities	Other	Provider

Hogan	2010	Hogan SO, Kissam SM. Measuring meaningful use. *Health Aff* 2010;**29**:601–6. doi:10.1377/hlthaff.2009.1023	1	Single country souce (non-EU)	USA	USA	National	Surveys	Non-continuous/ one off activity	Measuring eHealth/ict availability or use	Provider, HIE
Hyppönen	2013	Hyppönen H, Faxvaag A, Gilstad H, *et al.* Nordic eHealth indicators: organisation of research, first results and plan for the future. *Stud Health Technol Inform* 2013;**192**:273–7.	1	Multinational sources	Nordic countries	Northern Europe	National	Surveys	Non-continuous/ one off activity	Measuring eHealth/ict availability or use	Provider, Patient, HIE
Hyppönen	2013	Hyppönen H, Faxvaag A, Gilstad H, *et al.* Nordic eHealth Indicators. TemaNord: : Nordic Council of Ministers 2013. doi:10.6027/TN2013-522	2	Multinational sources	Nordic countries	Northern Europe	National	Surveys	Non-continuous/ one off activity	Measuring eHealth/ict availability or use	Provider, Patient, HIE
Hyppönen	2017	Hyppönen H, Kangas M, Reponen J, *et al.* Nordic eHealth Benchmarking - From piloting towards established practice. 2017. http://esc-web.lib.cbs.dk/login?url=http://dx.doi.org/10.6027/tn2015-539	2	Multinational sources	Nordic countries	Northern Europe	National	Surveys	Non-continuous/ one off activity	Measuring eHealth/ict availability or use	Patient
Hyppönen	2015	Hyppönen H, Kangas M, Reponen J, *et al.* Nordic eHealth Benchmarking - status 2014. 2015. http://esc-web.lib.cbs.dk/login?url=http://dx.doi.org/10.6027/tn2015-539	2	Multinational sources	Nordic countries	Northern Europe	National	Other	Non-continuous/ one off activity	Measuring eHealth/ict availability or use	Provider, Patient, HIE
Hyppönen	2015	Hyppönen H, Hämäläinen P, Reponen J. E-health and e-welfare of Finland. Tampere: 2015. http://urn.fi/URN:ISBN:978-952-302-563-9	2	Single country source (EU)	Finland	Northern Europe	National	Survey	Continouing data gathering activities	Measuring eHealth/ict availability or use	Provider, Patient, HIE
Jha	2009	Jha AK, DesRoches CM, Campbell EG, *et al.* Use of Electronic Health Records in U.S. Hospitals. *N Engl J Med* 2009;**360**:1628–38. doi:10.1056/NEJMsa0900592	1	Single country souce (non-EU)	USA	USA	National	Surveys	Continouing data gathering activities	Measuring eHealth/ict availability or use	Provider
Jaana	2012	Jaana M, Ward MM, Bahensky JA. EMRs and clinical is implementation in hospitals: A statewide survey. *J Rural Heal* 2012;**28**:34–43. doi:10.1111/j.1748-0361.2011.00386.x	1	Single country souce (non-EU)	USA	USA	Regional	Surveys	Non-continuous/ one off activity	Measuring eHealth/ict availability or use	Provider
Kim	2017	Kim YG, Jung K, Park YT, *et al.* Rate of electronic health record adoption in South Korea: A nation-wide survey. *Int J Med Inform* 2017;**101**:100–7. doi:10.1016/j.ijmedinf.2017.02.009	1	Single country souce (non-EU)	South Korea	Asia	National	Surveys	Other	Measuring eHealth/ict availability or use	Provider
Kushniruk	2013	Kushniruk A, Kaipio J, Nieminen M, *et al.* Comparing approaches to measuring the adoption and usability of electronic health records: Lessons learned from Canada, Denmark and Finland. *Stud Health Technol Inform* 2013;**192**:367–71. doi:10.3233/978-1-61499-289-9-367	1	Multinational sources	Canada, Denmark, Finland	Northern Europe, Canada	National	No data source	None	Measuring eHealth/ict availability or use	Provider
Liebe	2013	Liebe JD, Hübner U. Developing and trialling an independent, scalable and repeatable it-benchmarking procedure for healthcare organisations. *Methods Inf Med* 2013;**52**:360–9. doi:10.3414/ME12-02-0016	1	Single country source (EU)	Germany	Western Europe	National	Surveys	Non-continuous/ one off activity	Measuring eHealth/ict availability or use	Provider
Marca	2014	Marca G, Pérez AJ, Blanco-García MG, *et al.* The use of electronic health records in Spanish hospitals. *Heal Inf Manag J* 2014;**43**:37–44. doi:10.12826/18333575.2014.0003.Ortiga	1	Single country source (EU)	Spain	Southern Europe	National	Surveys	Non-continuous/ one off activity	Measuring eHealth/ict availability or use	Provider
Meyer	2009	Meyer I, Hüsing T, Didero M, *et al.* eHealth Benchmarking (Phase II) - Final report. Bonn: 2009. https://joinup.ec.europa.eu/sites/default/files/files_epractice/sites/eHealth Benchmarking (Phase II)-Final Report.pdf (accessed 5 Jan 2017).	3	No data	No data	No data	National	No data	Non-continuous/ one off activity	Measuring eHealth/ict availability or use	Provider, Patient, HIE
Nakamura	2013	Nakamura MM, Harper MB, Jha AK. Change in adoption of electronic health records by US children's hospitals. *Pediatrics* 2013;**131**:e1563-75. doi:10.1542/peds.2012-2904	1	Single country souce (non-EU)	USA	USA	National	Surveys	Continouing data gathering activities	Measuring eHealth/ict availability or use	Provider
OECD	2013	OECD, Directorate for Employment, Social Affairs, *et al.* Draft OECD guide to measuring ICRs in the health sector. 2013;:56.	3	No data	No data	No data	No data	No data	None	Measuring eHealth/ict availability or use	Defining the ICT domains
Palacio	2010	Palacio C, Harrison JP, Garets D, *et al.* Benchmarking electronic medical records initiatives in the US: A conceptual model. *J Med Syst* 2010;**34**:273–9. doi:10.1007/s10916-008-9238-5	1	Single country souce (non-EU)	USA	USA	National	Other	Continouing data gathering activities	Measuring eHealth/ict availability or use	Provider

Park	2017	Park Y-T, Han D. Current Status of Electronic Medical Record Systems in Hospitals and Clinics in Korea. *Healthc Inform Res* 2017;**23**:189–98. doi:10.4258/hir.2017.23.3.189	1	Single country souce (non-EU)	South Korea	Asia	National	Surveys	Non-continuous/ one off activity	Measuring eHealth/ict availability or use	Provider, HIE
Petersen	2015	Petersen LS, Bertelsen P, Tornberg K. Undersøgelse af borgernes perspektiv på sundheds-it i 2015 - en udforskning af danskernes kendskab, holdninger, anvendelse og forhold til it til gavn for eget helbred. Aalborg: 2015.	2	Single country source (EU)	Denmark	Northern Europe	National	Surveys	Continouing data gathering activities	Measuring eHealth/ict availability or use	Patient
PwC	2014	PwC. European Hospital Survey - Benchmarking Deployment of eHealth services (2012-2013). Luxembourg: : Publications Office of the European Union 2014. doi:10.2791/56790	2	Multinational sources	27 EU Member States and Croatia, Iceland and Norway.	Northern Europe, Western Europe, Eastern Europe, Southern Europe,	National	Surveys	Non-continuous/ one off activity	Measuring eHealth/ict availability or use	Provider, Patient, HIE
Singh	2012	Singh R, Lichter MI, Danzo A, *et al.* The adoption and use of health information technology in rural areas: Results of a national survey. *J Rural Heal* 2012;**28**:16–27. doi:10.1111/j. 1748-0361.2011.00370.x	1	Single country souce (non-EU)	USA	USA	National	Surveys	Non-continuous/ one off activity	Measuring eHealth/ict availability or use	Provider, HIE
Soderberg	2013	Soderberg K, Laventure M. Minnesota clinics' adoption, use and exchange of electronic health information. *Minn Med* 2013;**96**:45–8.	1	Single country souce (non-EU)	USA	USA	Regional	Surveys	Continouing data gathering activities	Measuring eHealth/ict availability or use	Provider, HIE
Thornberg	2014	Tornbjerg K, Nøhr C. Undersøgelse af klinisk anvendelse af sundheds-it-systemer 2014. Aalborg: 2014. http://dachi.dk/udgivelser/tech reports/2011/11_4 Kliniske anvendelse af sundheds it systemer 2010.pdf%5Cnhttp://vbn.aau.dk/ws/files/66744725/11_4_Kliniske_anvendelse_af_sundheds_it_systemer_2010.pdf	2	Single country source (EU)	Danmark	Northern Europe	National	Survey	Continouing data gathering activities	Measuring eHealth/ict availability or use	Provider
Viitanen	2011	Viitanen J, Hyppönen H, Laaveri T, *et al.* National questionnaire study on clinical ICT systems proofs: physicians suffer from poor usability. *Int J Med Inform* 2011;**80**:708–25. doi:10.1016/j.ijmedinf.2011.06.010	1	Single country source (EU)	Finland	Northern Europe	National	Surveys	Continouing data gathering activities	Measuring eHealth/ict availability or use	Provider
Villalba-Mora	2015	Villalba-Mora E, Casas I, Lupianez-Villanueva F, *et al.* Adoption of health information technologies by physicians for clinical practice: The Andalusian case. *Int J Med Inform* 2015;**84**:477–85. doi:10.1016/j.ijmedinf.2015.03.002	1	Single country source (EU)	Spain	Southern Europe	Regional	Surveys	Non-continuous/ one off activity	Measuring eHealth/ict availability or use	Provider, HIE
World Health Organisation	2016	WHO Global Observatory for eHealth. Atlas of eHealth country profiles. The use of eHealth in support of universal health coverage. *WHO, Geneva* 2016;:392.http://scholar.google.com/scholar?hl=en&btnG=Search&q=intitle:Atlas+of+eHealth+Country+Profiles#0	2	Multinational sources	125 WHO Member States	Northern Europe, Western Europe, Estern Europe, Southern Europe, Canada, USA, Asia, Other	National	Surveys	Continouing data gathering activities	Measuring eHealth/ict availability or use	Provider, Patient, HIE
World Health Organisation	2016	WHO Global Observatory for eHealth. Global diffusion of eHealth: Making universal health coverage achievable. Report of the third global survey on eHealth Global Observatory for eHealth. 2016. http://www.wipo.int/amc/en/mediation/rules	2	Multinational sources	125 WHO Member States	Northern Europe, Western Europe, Estern Europe, Southern Europe, Canada, USA, Asia, Other	National	Surveys	Continouing data gathering activities	Measuring eHealth/ict availability or use	Provider, Patient, HIE

Zelmer	2017	Zelmer J, Ronchi E, Hyppönen H, *et al.* International health IT benchmarking: Learning from cross-country comparisons. *J Am Med Informatics Assoc* 2017;**24**:371–9. doi:10.1093/jamia/ocw111	1	Multinational sources	38 countries: Brazil Israel Korea Uruguay Canada Denmark Finland Germany and Austria (acute care) The Netherlands Switzerland United Kingdom United States (Sweden, Norway, Iceland in the context of Nordic collaboration) Austria, Belgium, Bulgaria, Croatia, Cyprus, Czech Republic, Denmark, Estonia, Finland, France, Germany, Greece, Hungary, Iceland, Ireland, Italy, Latvia, Lithuania, Luxembourg, Malta, the Netherlands, Norway, Poland, Portugal, Romania, Slovakia, Slovenia, Spain, Sweden, Turkey (acute care survey only), United Kingdom	Northern Europe, Western Europe, Estern Europe, Southern Europe, Canada, USA, Asia, Other	National	Surveys	Non-continuous/ one off activity	Measuring eHealth/ict availability or use	Provider, Patient, HIE
